# Supplementary material for: Alcohol and HIV-Derived Hepatocyte Apoptotic Bodies Induce Hepatic Stellate Cell Activation
Source: Biology (Basel). 2022 Jul 14;11(7):1059. doi: 10.3390/biology11071059 (PMC9312505; doi:10.3390/biology11071059)

All western blots

Figure S2D

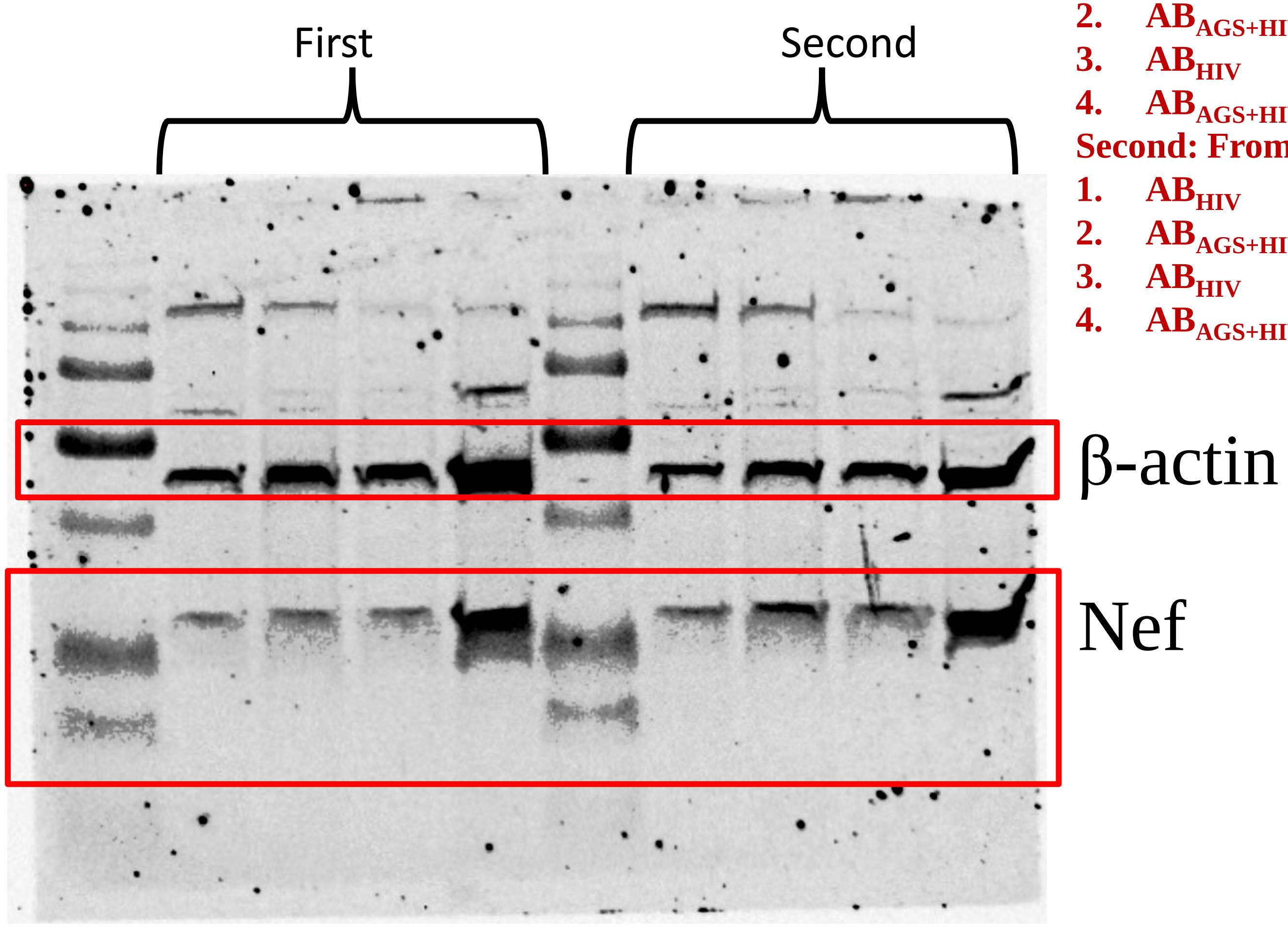

First: From left to right

1. AB_{HIV}
2. $AB_{AGS+HIV}$
3. AB_{HIV}
4. $AB_{AGS+HIV}$

Second: From left to right

1. AB_{HIV}
2. $AB_{AGS+HIV}$
3. AB_{HIV}
4. $AB_{AGS+HIV}$

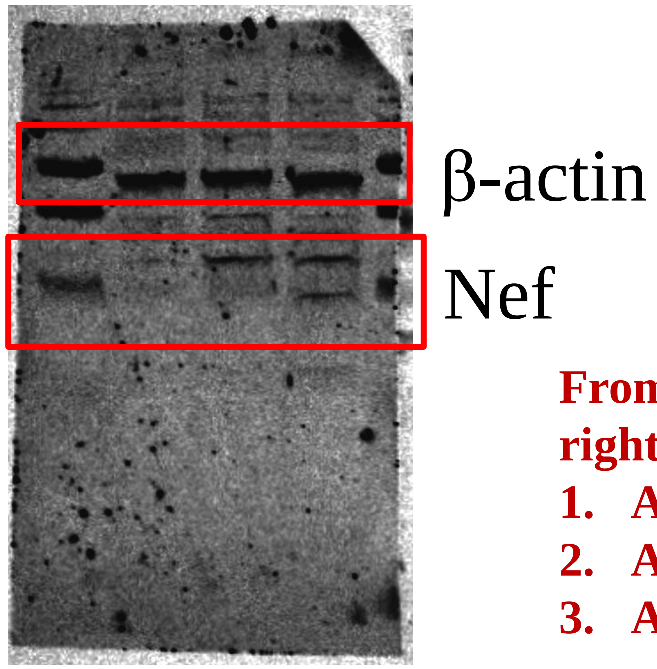

From left to right

1. $AB_{Control}$
2. AB_{HIV}
3. $AB_{AGS+HIV}$

Figure S2D

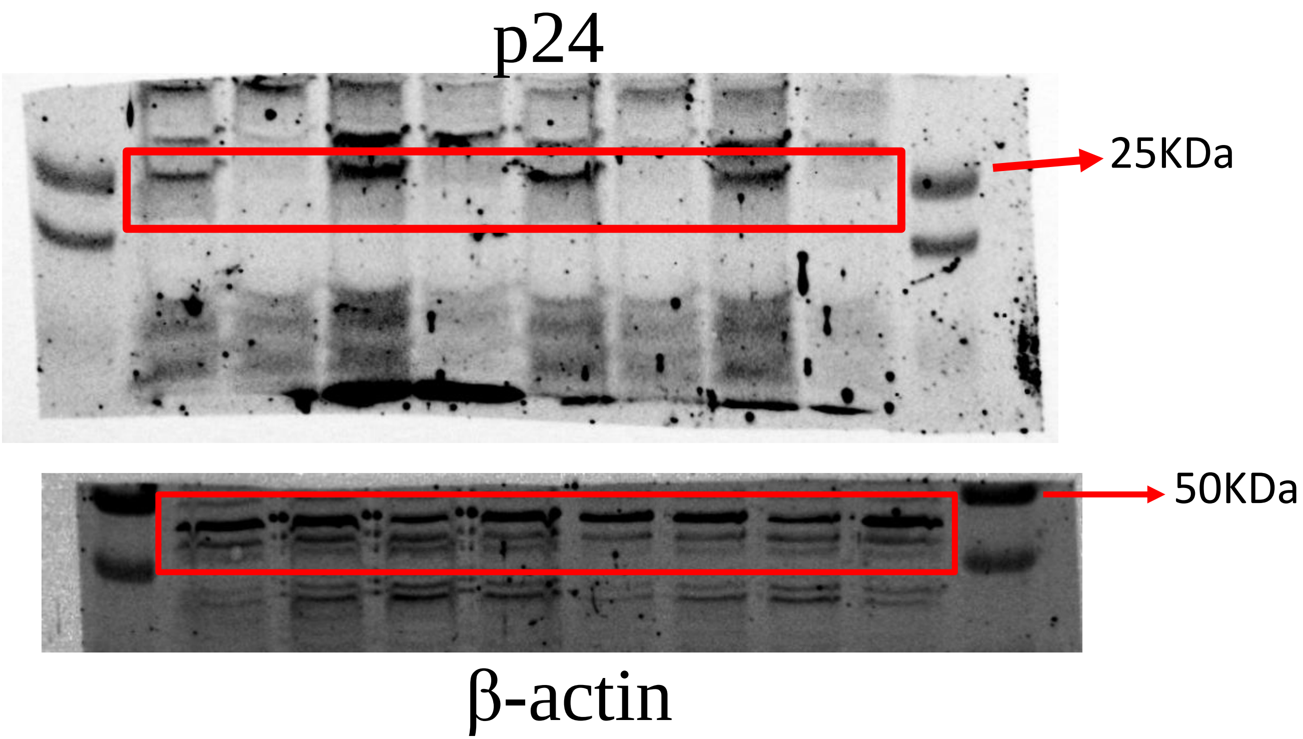

From left to right lanes

1. AB$_{HIV}$
2. AB$_{Control}$
3. AB$_{AGS+HIV}$
4. AB$_{AGS}$
5. AB$_{HIV}$
6. AB$_{Control}$
7. AB$_{AGS+HIV}$
8. AB$_{AGS}$

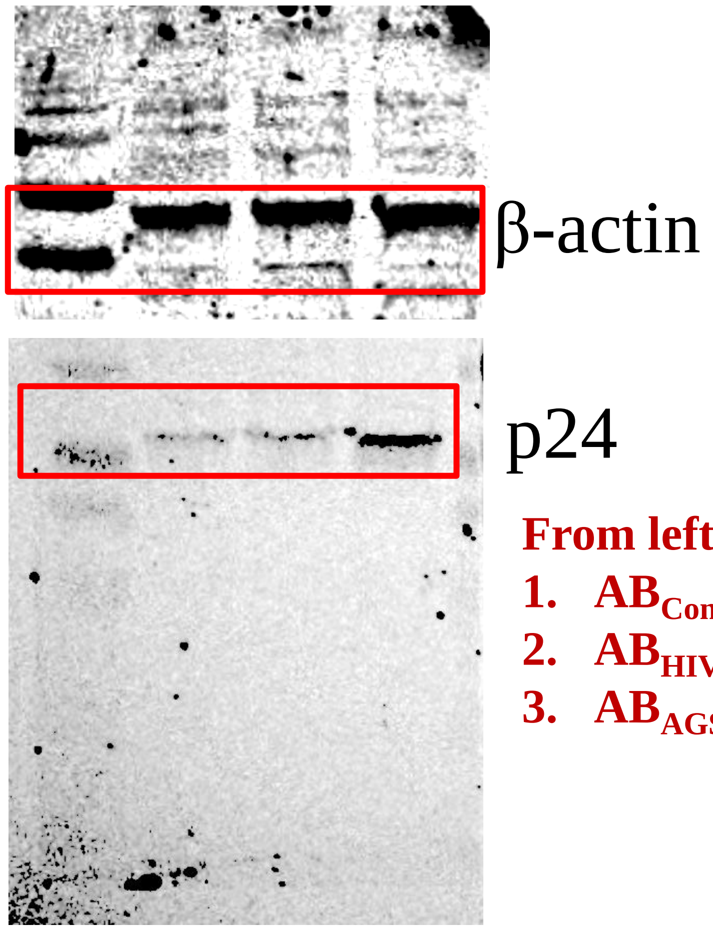

From left to right

1. AB$_{Control}$
2. AB$_{HIV}$
3. AB$_{AGS+HIV}$

Figure S2D

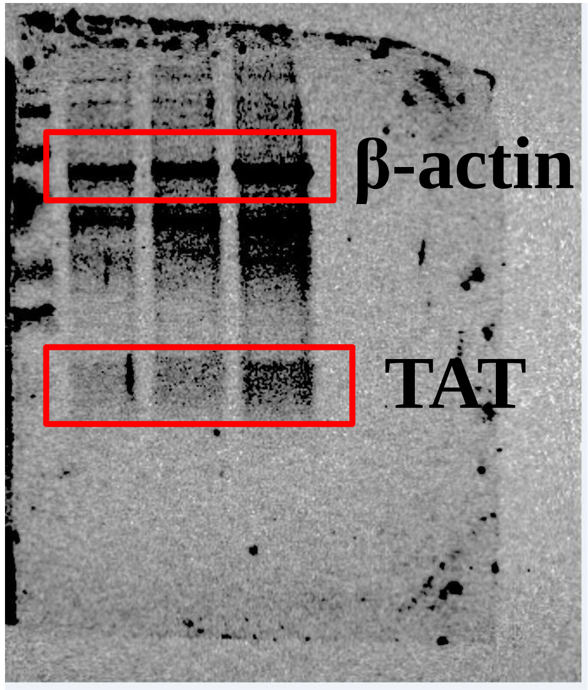

From left to Right

AB$_{Control}$

AB$_{HIV}$

AB$_{AGS+HIV}$

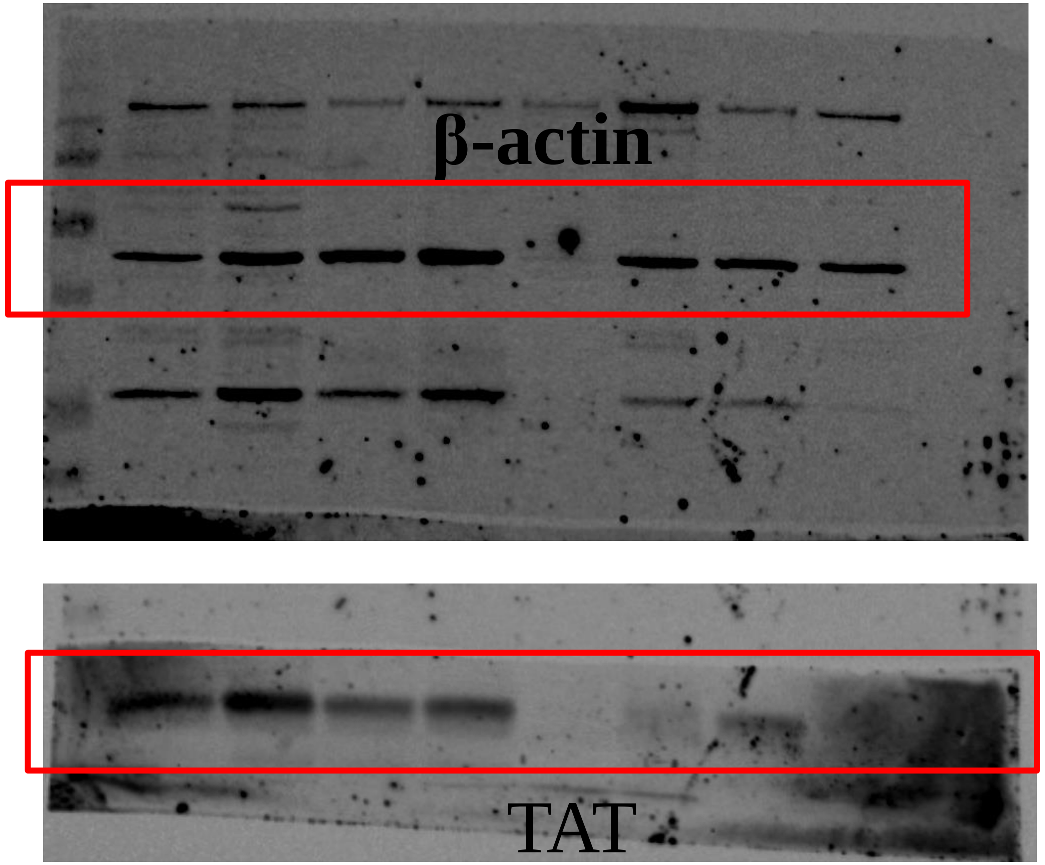

From left to Right

1. **AB$_{AGS+HIV}$**
2. **AB$_{AGS+HIV}$**
3. **AB$_{HIV}$**
4. **AB$_{HIV}$**
5. **Gap**
6. **AB$_{Contol}$**
7. **AB$_{HIV}$**
8. **Canceled**

Figure S2H

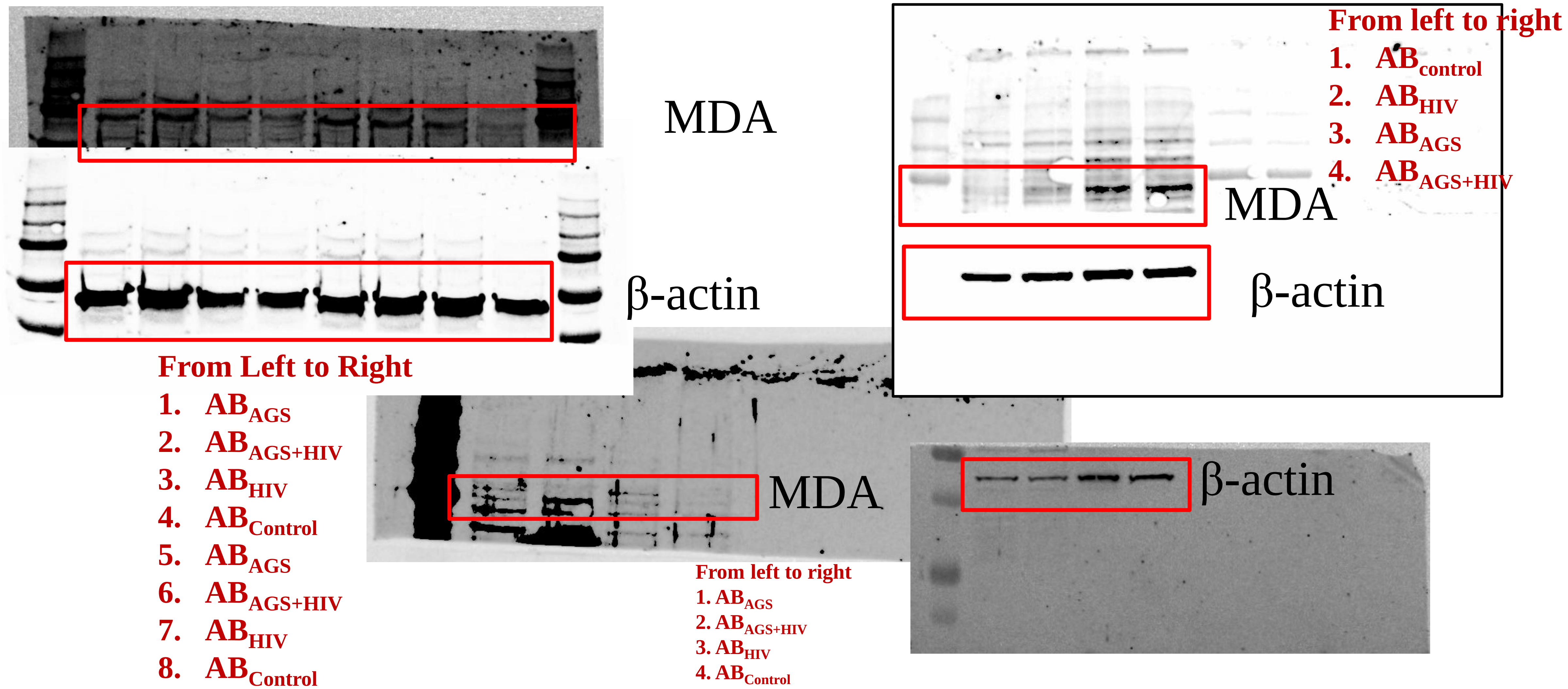
MDA
β-actin
From Left to Right
1. AB_{AGS}
2. $AB_{AGS+HIV}$
3. AB_{HIV}
4. $AB_{Control}$
5. AB_{AGS}
6. $AB_{AGS+HIV}$
7. AB_{HIV}
8. $AB_{Control}$
From left to right
1. $AB_{control}$
2. AB_{HIV}
3. AB_{AGS}
4. $AB_{AGS+HIV}$
MDA
β-actin
MDA
β-actin
From left to right
1. AB_{AGS}
2. $AB_{AGS+HIV}$
3. AB_{HIV}
4. $AB_{Control}$

Figure S2J

From left to right

1. **RLW Whole cell lysate**
2. **$AB_{Control}$**
3. **AB_{HIV}**
4. **AB_{AGS}**
5. **$AB_{AGS+HIV}$**

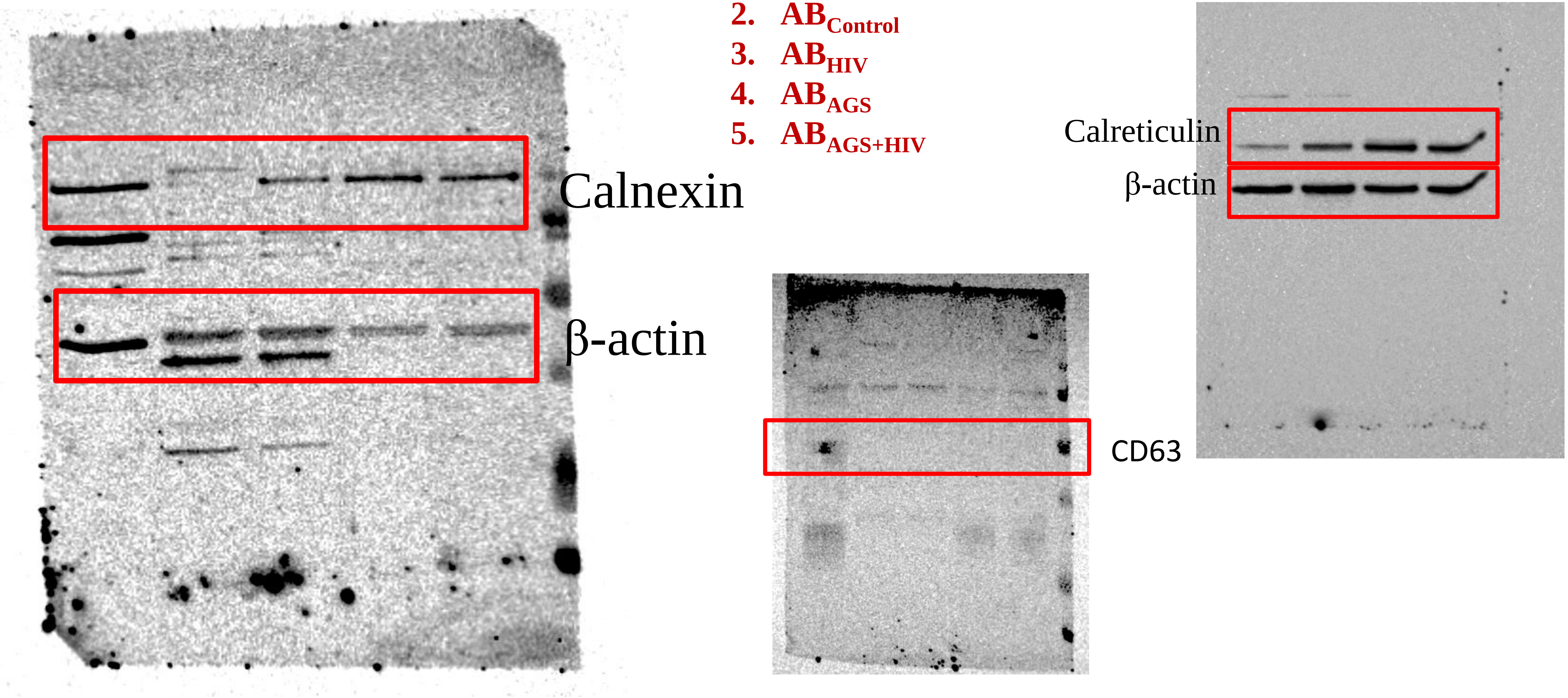

Figure S5I

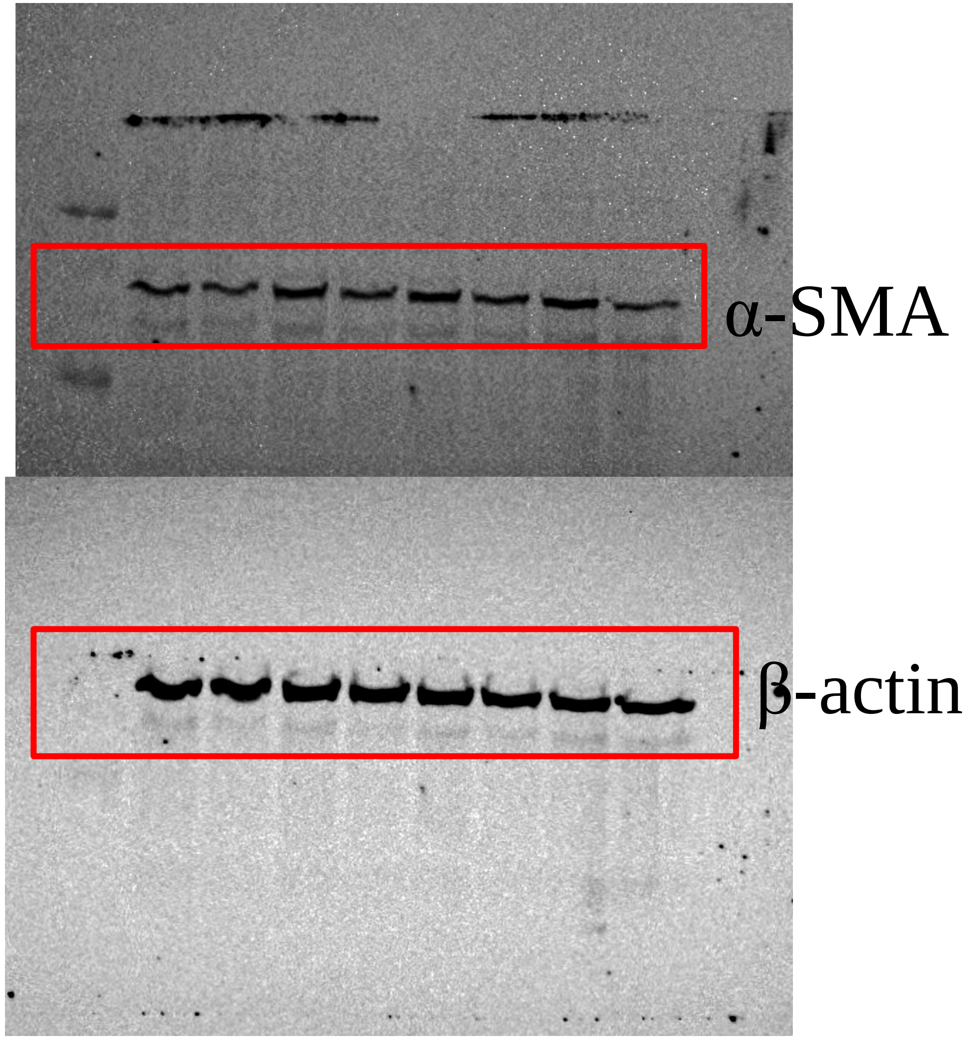

From left to right

1. **LX2+AB$_{Control}$**
2. **LX2+AB$_{Control}$**
3. **LX2+AB$_{AGS+HIV}$**
4. **LX2+AB$_{Control}$**
5. **LX2+AB$_{AGS+HIV}$**
6. **LX2+AB$_{Control}$**
7. **LX2+AB$_{AGS+HIV}$**
8. **LX2+AB$_{Control}$**

Figure S6D

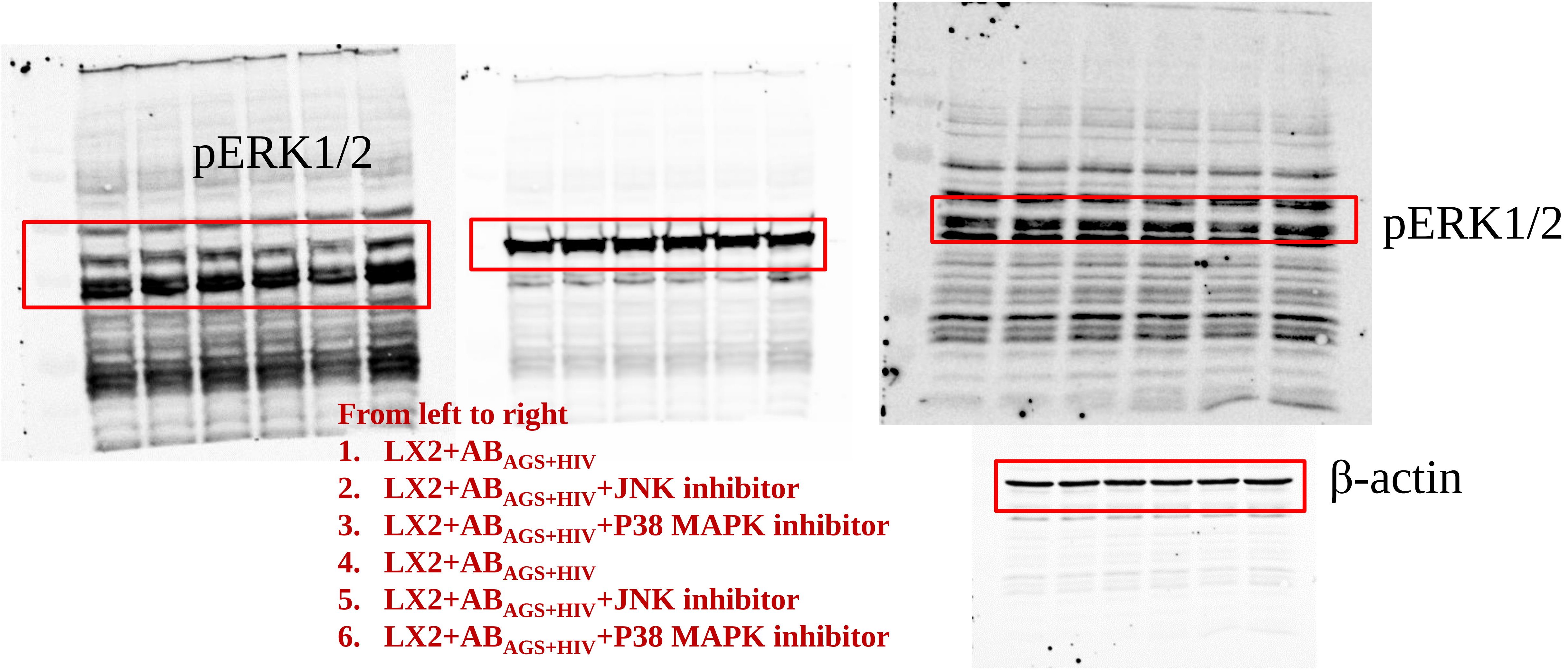
pERK1/2
pERK1/2
β-actin
From left to right
1. LX2+AB$_{AGS+HIV}$
2. LX2+AB$_{AGS+HIV}$+JNK inhibitor
3. LX2+AB$_{AGS+HIV}$+P38 MAPK inhibitor
4. LX2+AB$_{AGS+HIV}$
5. LX2+AB$_{AGS+HIV}$+JNK inhibitor
6. LX2+AB$_{AGS+HIV}$+P38 MAPK inhibitor

Figure S6D

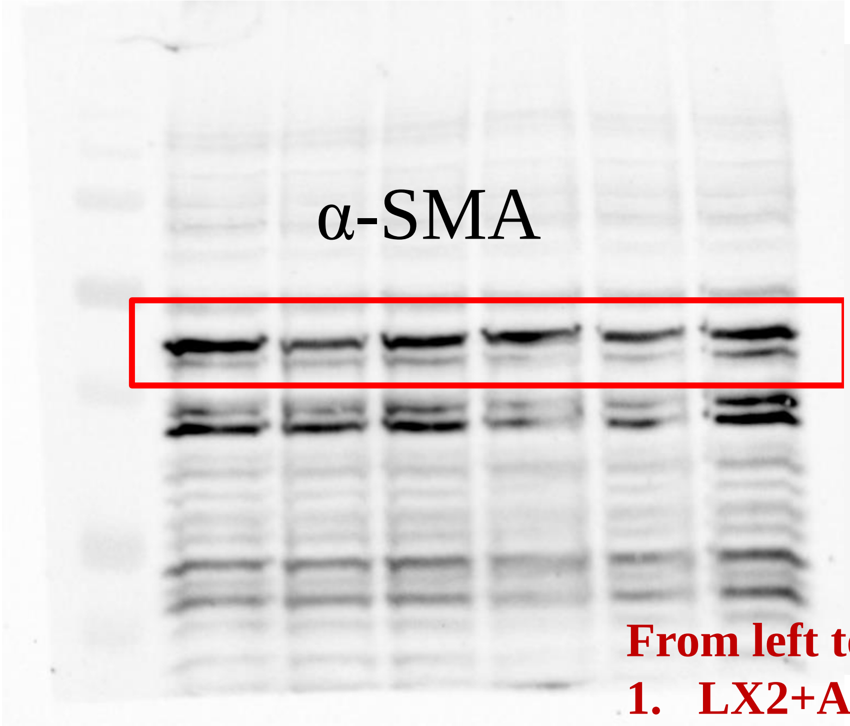

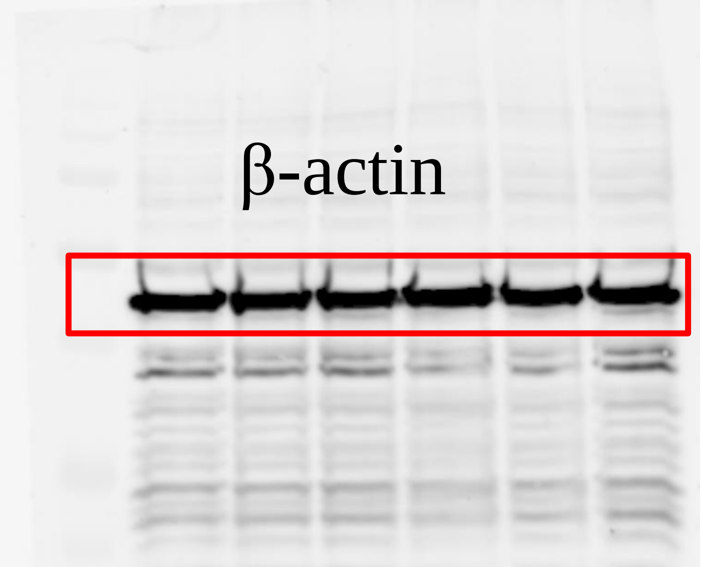

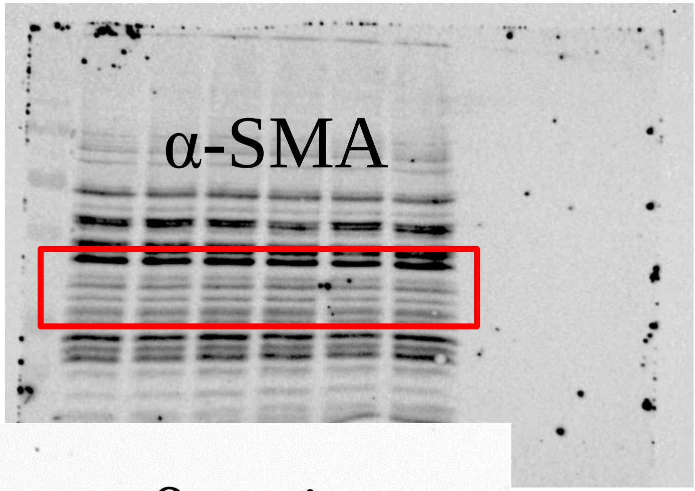

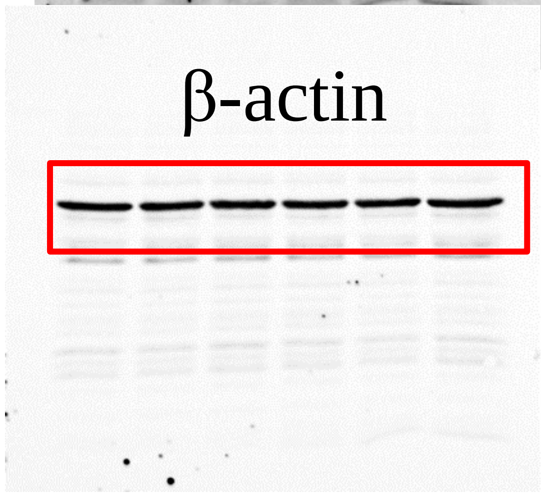

From left to right

1. **LX2+$AB_{AGS+HIV}$**
2. **LX2+$AB_{AGS+HIV}$+JNK inhibitor**
3. **LX2+$AB_{AGS+HIV}$+P38 MAPK inhibitor**
4. **LX2+$AB_{AGS+HIV}$**
5. **LX2+$AB_{AGS+HIV}$+JNK inhibitor**
6. **LX2+$AB_{AGS+HIV}$+P38 MAPK inhibitor**

Figure S6D

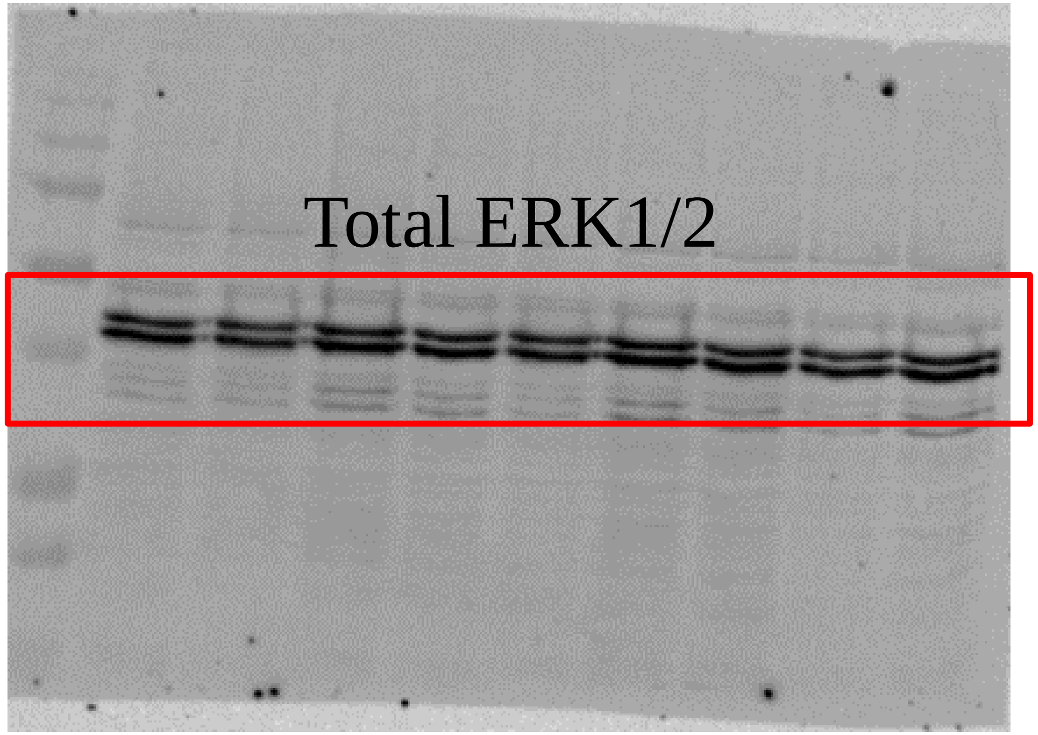

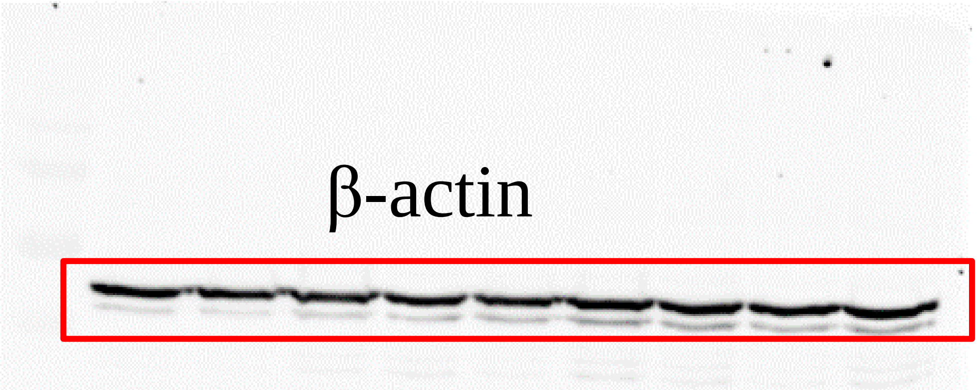

From left to right

1. **1. LX2+$AB_{AGS+HIV}$**
2. **LX2+$AB_{AGS+HIV}$+JNK inhibitor**
3. **LX2+$AB_{AGS+HIV}$+P38 MAPK inhibitor**
4. **LX2+$AB_{AGS+HIV}$**
5. **LX2+$AB_{AGS+HIV}$+JNK inhibitor**
6. **LX2+$AB_{AGS+HIV}$+P38 MAPK inhibitor**
7. **LX2+$AB_{AGS+HIV}$**
8. **LX2+$AB_{AGS+HIV}$+JNK inhibitor**
9. **LX2+$AB_{AGS+HIV}$+P38 MAPK inhibitor**

Figure S7C

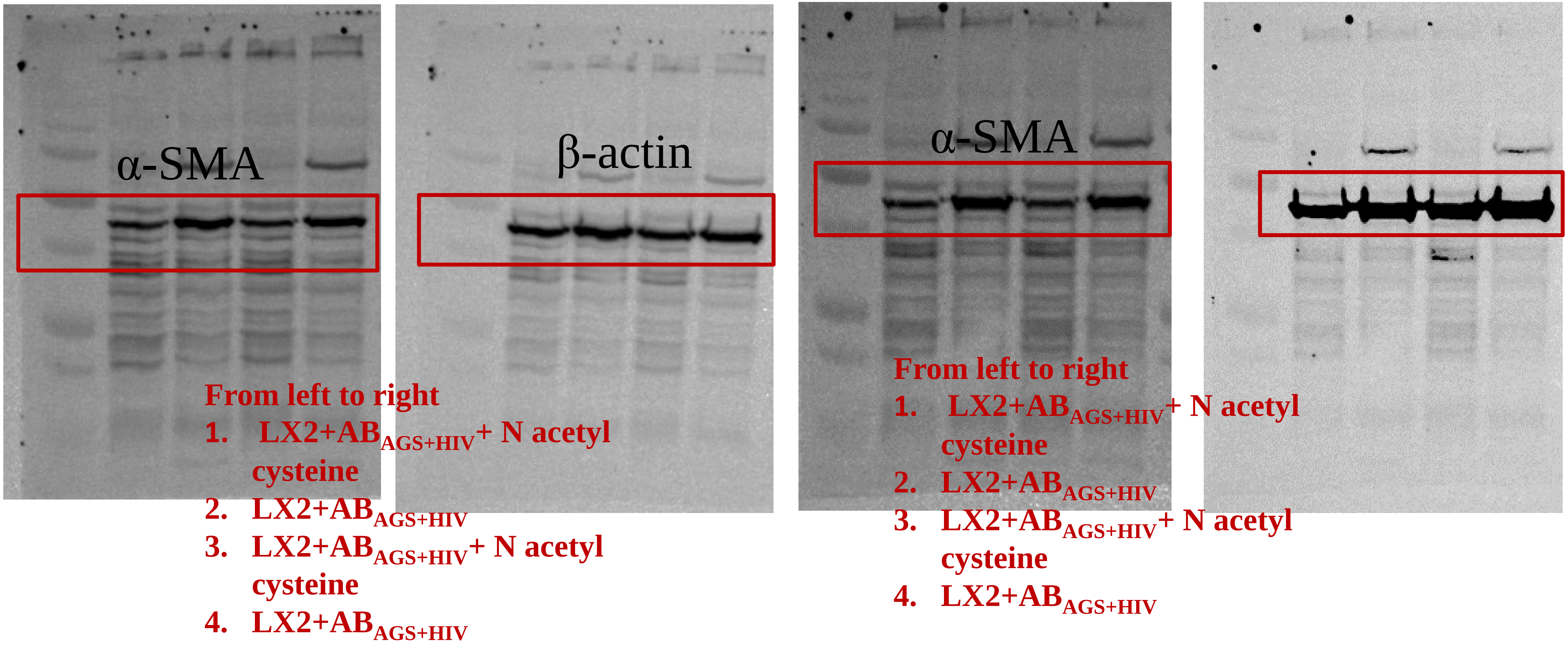

α-SMA
β-actin
From left to right
1. LX2+AB$_{AGS+HIV}$+ N acetyl cysteine
2. LX2+AB$_{AGS+HIV}$
3. LX2+AB$_{AGS+HIV}$+ N acetyl cysteine
4. LX2+AB$_{AGS+HIV}$
α-SMA
From left to right
1. LX2+AB$_{AGS+HIV}$+ N acetyl cysteine
2. LX2+AB$_{AGS+HIV}$
3. LX2+AB$_{AGS+HIV}$+ N acetyl cysteine
4. LX2+AB$_{AGS+HIV}$

Figure S7C

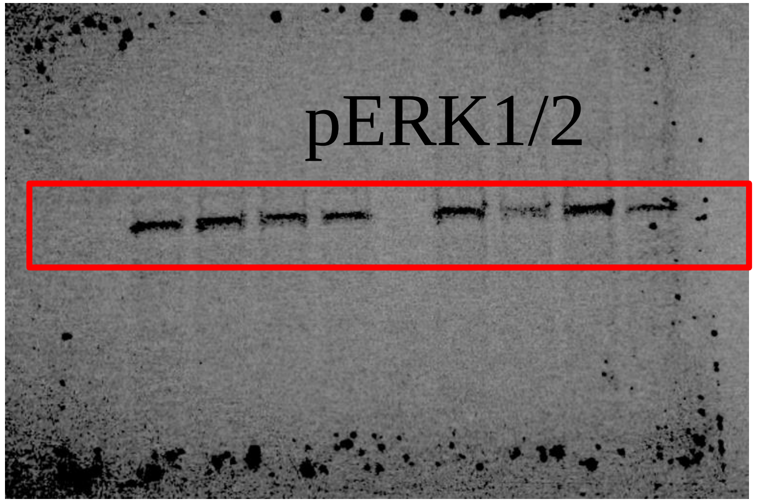

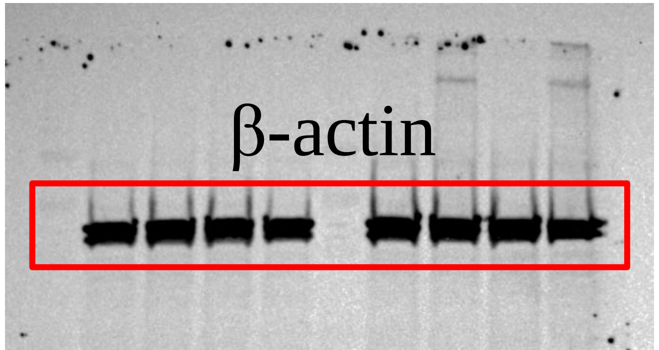

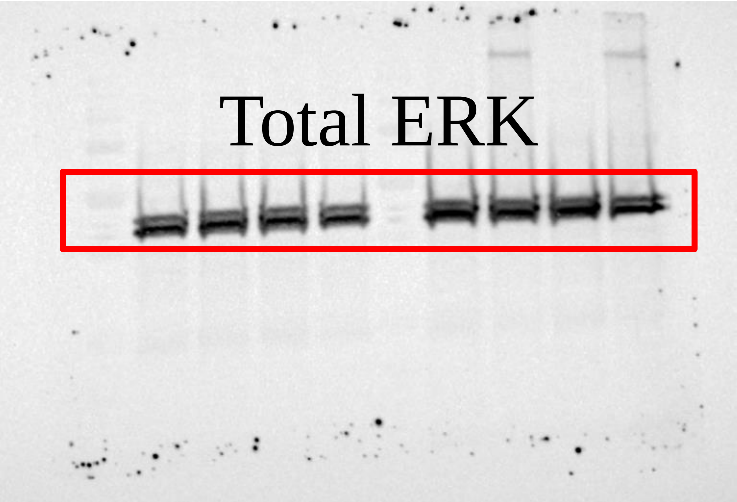

From left to right

1. **LX2+AB$_{AGS+HIV}$**
2. **LX2+AB$_{AGS+HIV}$**
3. **LX2+AB$_{AGS+HIV}$+N acetyl cysteine**
4. **LX2+AB$_{AGS+HIV}$+ N acetyl cysteine**
5. **GAP**
6. **LX2+AB$_{AGS+HIV}$**
7. **LX2+AB$_{AGS+HIV}$+N acetyl cysteine**
8. **LX2+AB$_{AGS+HIV}$**
9. **LX2+$_{ABAGS+HIV}$+ N acetyl cysteine**

Figure S8A

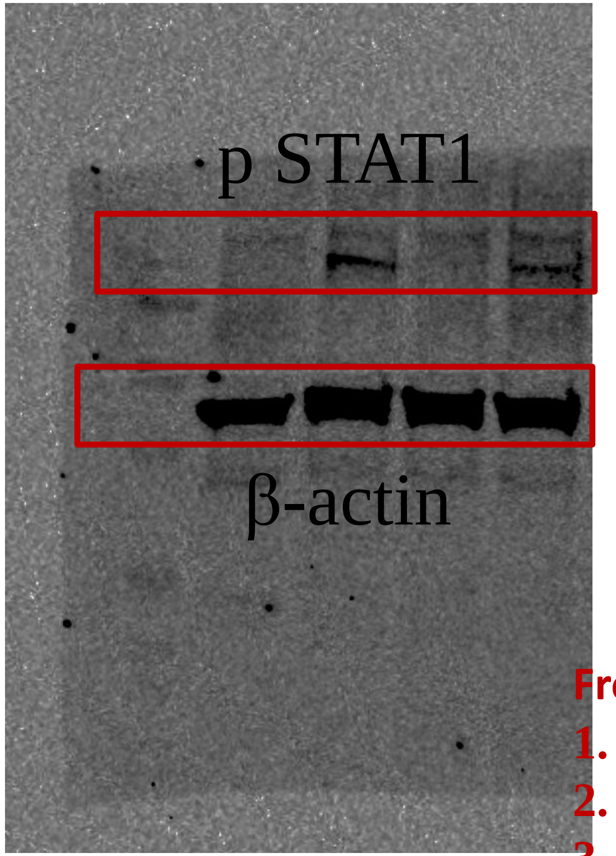

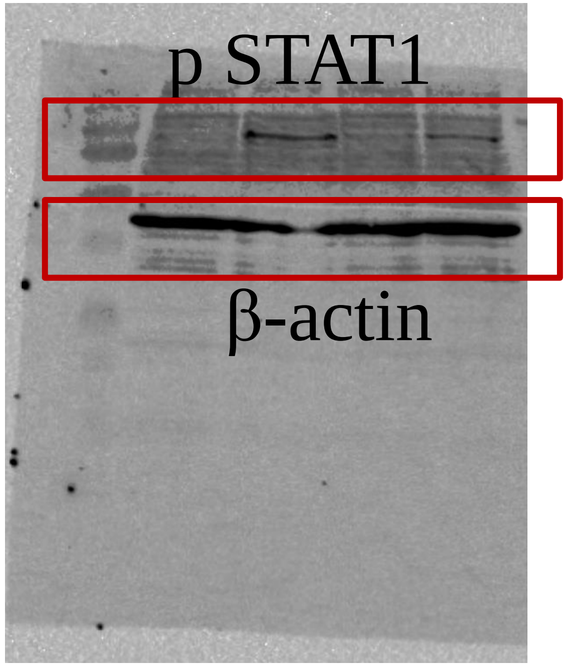

From left to right

1. LX2
2. LX2+IFN
3. LX2+AB$_{AGS+HIV}$
4. LX2+AB$_{AGS+HIV}$+IFN

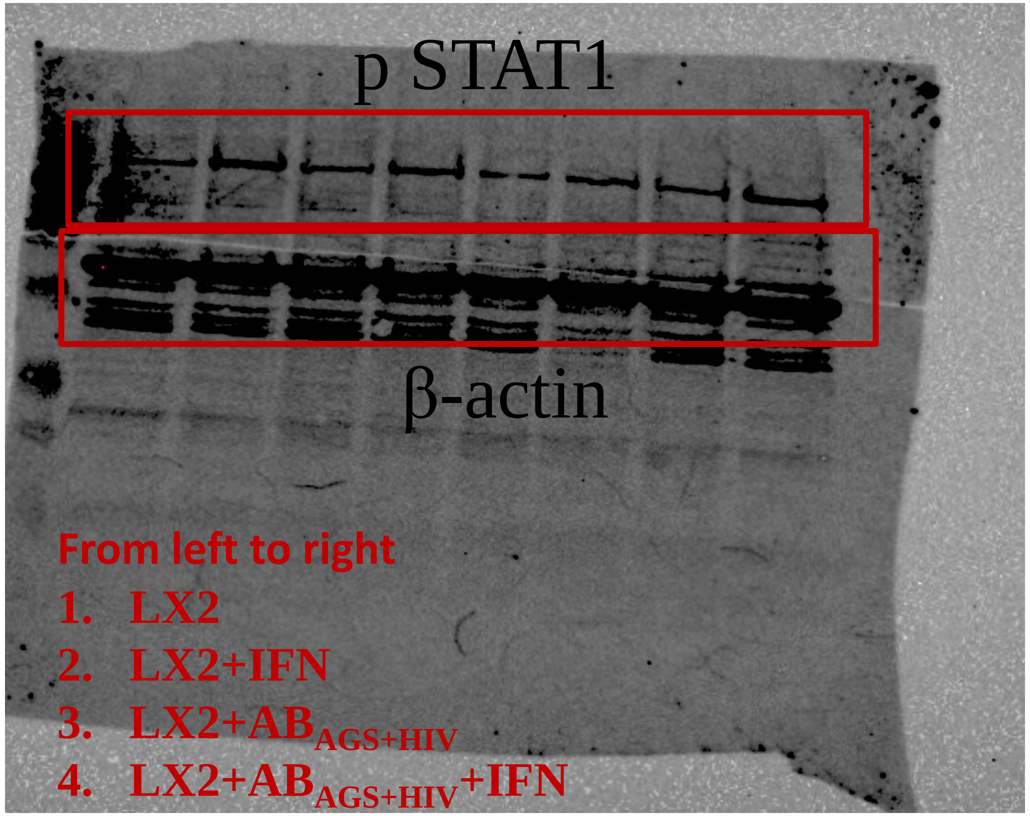

From left to right

1. LX2
2. LX2+IFN
3. LX2+AB$_{AGS+HIV}$
4. LX2+AB$_{AGS+HIV}$+IFN
5. LX2
6. LX2+IFN
7. LX2+AB$_{AGS+HIV}$
8. LX2+AB$_{AGS+HIV}$+IFN

Figure S8A

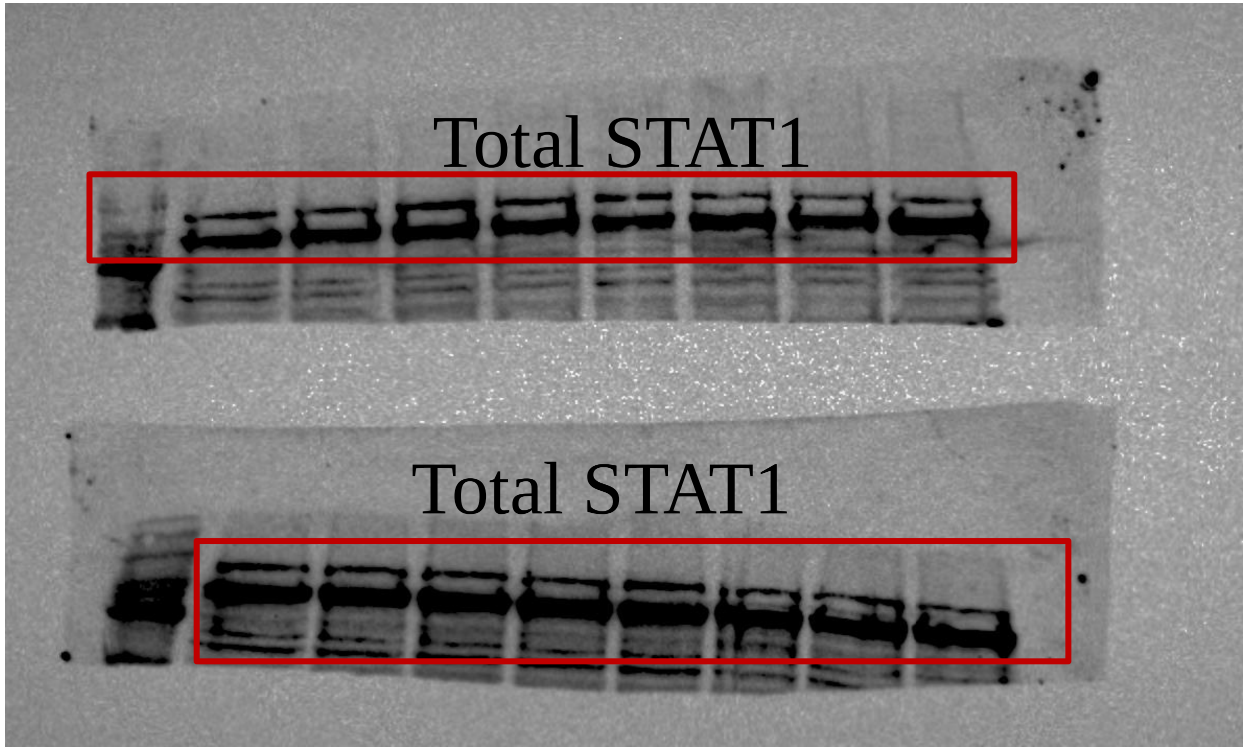

From left to right

1. **LX2**
2. **LX2+IFN**
3. **LX2+AB$_{AGS+HIV}$**
4. **LX2+AB$_{AGS+HIV}$+IFN**
5. **LX2**
6. **LX2+IFN**
7. **LX2+AB$_{AGS+HIV}$**
8. **LX2+AB$_{AGS+HIV}$+IFN**

Figure S9E and S10E

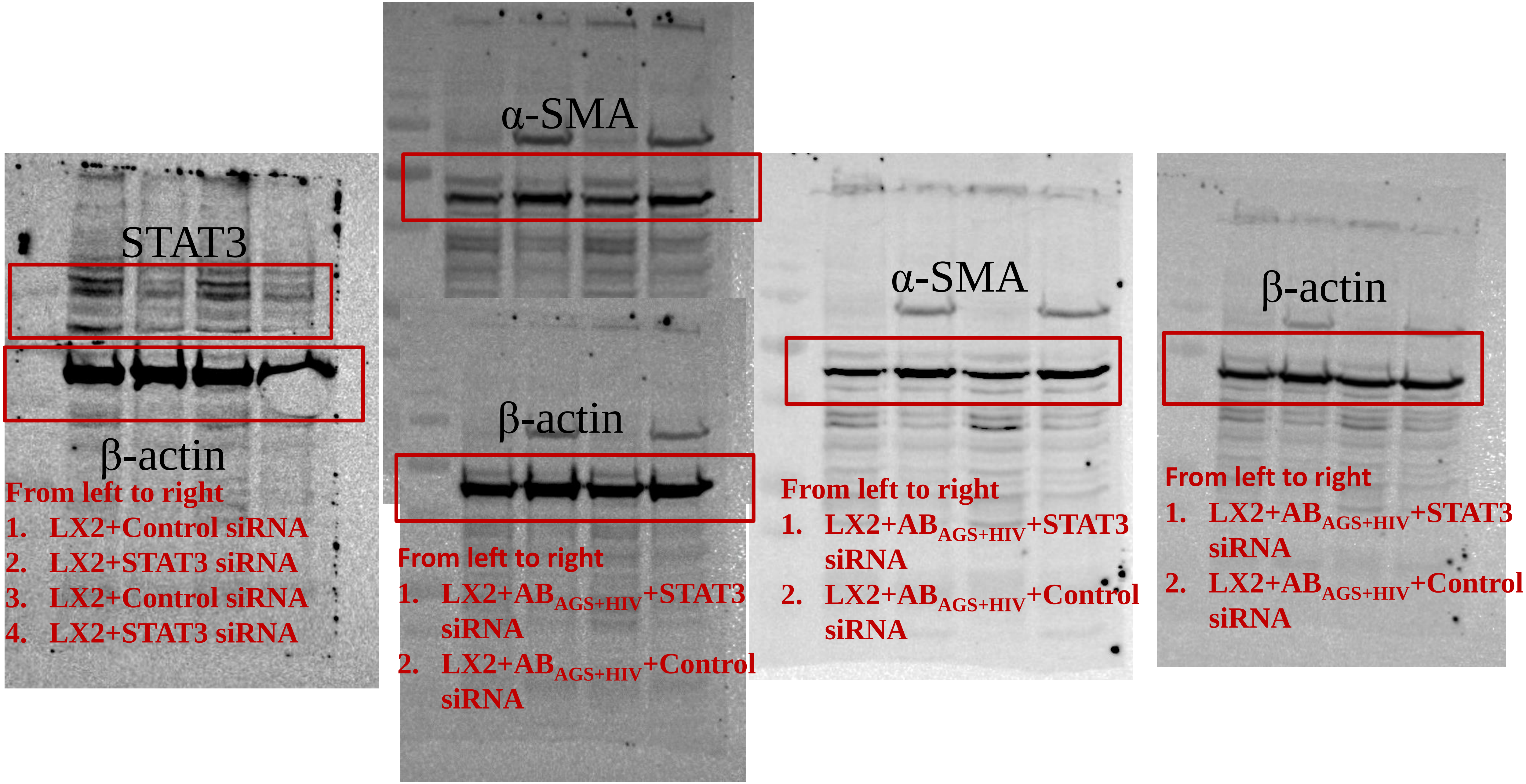

Supplement: Supplementary file 1 [file biology-11-01059-s001.zip › biology-1796964-supplementary.pdf]
